# The Human Mixed Lineage Leukemia 5 (MLL5), a Sequentially and Structurally Divergent SET Domain-Containing Protein with No Intrinsic Catalytic Activity

**DOI:** 10.1371/journal.pone.0165139

**Published:** 2016-11-03

**Authors:** Sarah Mas-y-Mas, Marta Barbon, Catherine Teyssier, Hélène Déméné, João E. Carvalho, Louise E. Bird, Andrey Lebedev, Juliana Fattori, Michael Schubert, Christian Dumas, William Bourguet, Albane le Maire

**Affiliations:** 1 Inserm U1054, Centre de Biochimie Structurale, Montpellier, France; 2 CNRS UMR5048, Centre de Biochimie Structurale, Montpellier, France; 3 Université de Montpellier, Montpellier, France; 4 IRCM, Institut de Recherche en Cancérologie de Montpellier, Montpellier, France; 5 Sorbonne Universités, UPMC Université Paris 06, CNRS, Laboratoire de Biologie du Développement de Villefranche-sur-Mer, Observatoire Océanologique de Villefranche-sur-Mer, Villefranche-sur-Mer, France; 6 OPPF-UK, Research Complex at Harwell, Rutherford Appleton Laboratory, Oxfordshire, OX11 0FA, United Kingdom; 7 CCP4, Research Complex at Harwell, Rutherford Appleton Laboratory, Oxfordshire, OX11 0FA, United Kingdom; 8 Centro Nacional de Pesquisa em Energia e Materiais, Laboratório Nacional de Biociências, Campinas, SP, Brazil; Universität Stuttgart, GERMANY

## Abstract

Mixed Lineage Leukemia 5 (MLL5) plays a key role in hematopoiesis, spermatogenesis and cell cycle progression. Chromatin binding is ensured by its plant homeodomain (PHD) through a direct interaction with the N-terminus of histone H3 (H3). In addition, MLL5 contains a Su(var)3-9, Enhancer of zeste, Trithorax (SET) domain, a protein module that usually displays histone lysine methyltransferase activity. We report here the crystal structure of the unliganded SET domain of human MLL5 at 2.1 Å resolution. Although it shows most of the canonical features of other SET domains, both the lack of key residues and the presence in the SET-I subdomain of an unusually large loop preclude the interaction of MLL5 SET with its cofactor and substrate. Accordingly, we show that MLL5 is devoid of any *in vitro* methyltransferase activity on full-length histones and histone H3 peptides. Hence, the three dimensional structure of MLL5 SET domain unveils the structural basis for its lack of methyltransferase activity and suggests a new regulatory mechanism.

## Introduction

*MLL5* was initially identified as a candidate tumor suppressor gene located in the commonly deleted 2.5 Mb segment of human chromosome band 7q22 in myeloid malignancies [1]. Knock-out studies in mice showed that the murine MLL5 protein is required in adult hematopoiesis [2–4] and normal spermatogenesis [5]. Overexpression and knockdown of MLL5 both induce cell cycle arrest at various phases, suggesting a versatile function of MLL5 throughout the cell cycle [6]. A recent study showed that MLL5 associates with chromatin regions downstream of transcriptional start sites of active genes and provided insights into the regulation of this association during mitosis and development [7].

Full-length MLL5 is a 205 kDa protein (1858 residues), although a smaller isoform (around 75 kDa) including a single Plant HomeoDomain (PHD) and a Su(var)3-9, Enhancer of zeste, Trithorax (SET) domain has been described [1]. The C-terminal region of MLL5, truncated in the shorter isoform, displays no apparent homology to any known structural domain and is predicted to be disordered in solution. The MLL5 protein is well conserved between species, with the human sequences for example, showing 80% and 38% identity with the PHD and SET domains of its *Drosophila* counterpart UpSET, respectively. Interestingly, both the *Drosophila* UpSET and the Yeast ortholog SET3, which are found in histone deacetylase complexes, were shown to contain inactive SET domains [8,9]. Even if a hallmark of SET domain-containing proteins is the histone methyltransferase (HMT) activity, it remains controversial whether the human and mouse MLL5 SET domains possess a catalytic HMT activity [2,10,11]. To better understand the molecular mechanism of human MLL5 function, we solved the crystal structure of its SET domain at 2.1 Å resolution. Although some common characteristics were observed, our structure reveals significant differences with canonical SET domains endowed with catalytic activity. Moreover, a correlative analysis of 3D structures and sequences allowed us to identify MLL5 specific features unfavorable for methyltransferase activity. Accordingly, biochemical and biophysical experiments showed that the SET domain of MLL5 is devoid of any binding activity towards histone peptides or the cofactor S-Adenosyl Methionine (SAM) *in vitro* and does not exhibit HMT activity on full-length histones. Having provided compelling evidence that MLL5 is not a functioning enzyme, the biological role of this protein remains to be established.

## Results

### The MLL5 SET domain lacks most of the residues important for HMT activity

**Fig 1:**
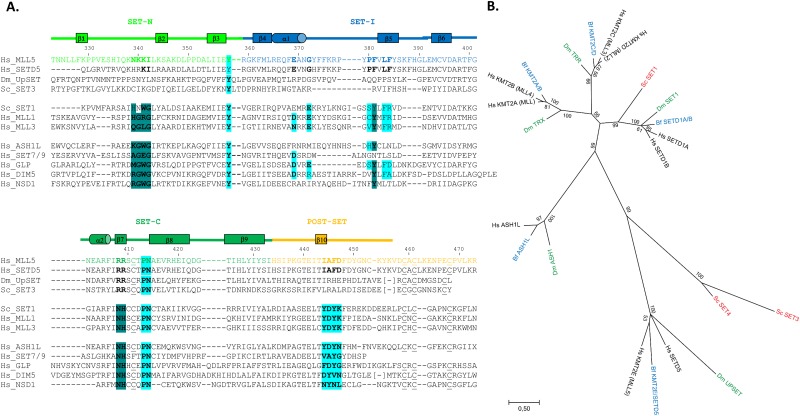
MLL5 SET domain lacks important residues for methyltransferase activity. **(A)**. Sequence alignment of the human MLL5 SET domain with SET domains from other methyltransferases (human SETD5, *Drosophila* UPSET, yeast SET3, yeast SET1, human MLL1, human MLL3, human ASH1L, human SET7/9, human GLP, human DIM5 and human NSD1). Structural alignment was calculated using the Dali server [56] when structure of the SET domain were available. Protein subdomains, indicated above the sequences, are based on the human MLL1 SET domain [14]. Secondary structure elements, derived from the structure of the MLL5 SET domain, are also shown above the sequences. Residues usually important for cofactor binding (dark blue) and for substrate binding and maintenance in the active center (cyan) are highlighted. Conserved cysteines from the zinc-binding cage are underlined. **(B)**. Phylogenetic tree of methyltransferases related to MLL5 using sequences from humans (*Homo sapiens*) (Hs, black), the cephalochordate amphioxus (*Branchiostoma floridae*) (Bf, blue), the fruit fly (*Drosophila melanogaster*) (Dm, green) and the yeast (*Saccharomyces cerevisiae*) (Sc, red). The tree resulting from the Maximum Likelihood (ML) analysis is shown and branch lengths are representative of sequence substitution rates. Branch support is indicated as Bootstrap percentages (ranging from 0 to 100) and as posterior probabilities (ranging from 0 to 1) obtained from the Bayesian Inference (BI) analysis (provided in parentheses at each node). “(—)” indicates that the branching patterns of the ML and BI analyses diverged at this node and that a posterior probability score is thus unavailable.

The MLL5 protein contains a SET domain (residues 323 to 433) followed by a POSTSET domain (residues 434 to 473). The prominent feature of the POSTSET domain is a zinc-binding cage formed from three cysteine residues of the C-terminal region with a fourth cysteine provided by the SET-C subdomain (Fig 1A). A common feature of SET domains is a channel through the protein linking the cofactor (SAM) binding surface on one side with the substrate binding surface on the other [12]. This channel, made up of residues from the SET and POSTSET regions, encloses the lysine residue of the substrate and holds it in an appropriate chemical environment and position for methyl transfer to take place (S1 Fig).

Sequence analysis of the MLL5 SET domain suggests that it does not contain all the conserved sequence elements required for methyltransferase activity. Contrary to other SET-domain containing methyltransferases, MLL5 does not have the residues usually involved in cofactor binding (Fig 1A). Instead of the highly conserved XGXG, Y and NH motifs, MLL5 displays NKKI (Asn 339-Ile 342), F (Phe 381) and RR (Arg 408—Arg 409) motifs. Key residues involved in histone H3 recognition and in the active center are also poorly conserved in MLL5 (Fig 1A). In particular, of the three conserved tyrosine residues in the active site of SET domains, two are replaced by other residues (Ile 444 and Phe 446 in MLL5). These substitutions are very likely to either significantly reduce the catalytic activity or to affect the mono, di- or tri-methylation specificity of the MLL5 SET domain [13]. Altogether, these important sequence differences strongly suggest that the MLL5 SET domain displays altered functional properties, when compared to SET domains of other proteins.

### The MLL5 protein is definitively not a member of the MLL family

In mammals, the Mixed Lineage Leukemia (MLL) family of SET domain-containing methyltransferases includes at least six members (MLL1, MLL2, MLL3, MLL4, SETD1A and SETD1B), all of which are involved in gene activation [14]. All members of this family share a distinct SET domain along with various numbers of PHD domains and catalyze the addition of methyl groups to the ε-amino moiety of lysine. Importantly, MLL family proteins are highly specific for lysine 4 of histone H3, which is mediated by their SET domain [15]. MLL5 was originally included in this MLL family due to its sequence conservation with the SET domain of MLL1 [1], although it was always considered as a distantly related member. Our phylogenetic analysis of human, *Drosophila*, yeast and amphioxus sequences shows that MLL5 and SETD5 form a family that is clearly distinct from the MLL1-4, SETD1A, B group (S2 Fig, Fig 1B). Moreover, while yeast SET1 is very closely related to human SETD1A, B and thus to human MLL1-4, yeast SET3 and SET4 are orthologs of human MLL5 and SETD5. Similarly, *Drosophila* UpSET belongs to the MLL5/SETD5 family. The presence of MLL5/SETD5 proteins in yeast, flies and humans indicates that this family is evolutionarily very ancient and was already present in the last common ancestor of fungi and animals (Fig 1B).

Like MLL5, yeast SET3 and SET4 as well as *Drosophila* UpSET also lack the XGXG, Y and NH catalytic sequence signatures present in MLL1-4 (Fig 1A) [15], suggesting that all members of the MLL5/SETD5 clade are characterized by a selective loss of catalytic activity. This notion is supported by functional analyses of yeast SET3 and *Drosophila* UpSET, which demonstrated that both proteins lack methyltransferase activity [8,9]. Although the catalytic capacities of yeast SET4 and human SETD5 remain to be determined, it is very likely that the loss of catalytic activity is characteristic of all MLL5 and SETD5 sequences and thus took place soon after the evolutionary origin of this family.

### The isolated MLL5 SET domain is not able to bind the S-adenosyl-methionine and the H3K4 histone peptide

**Fig 2:**
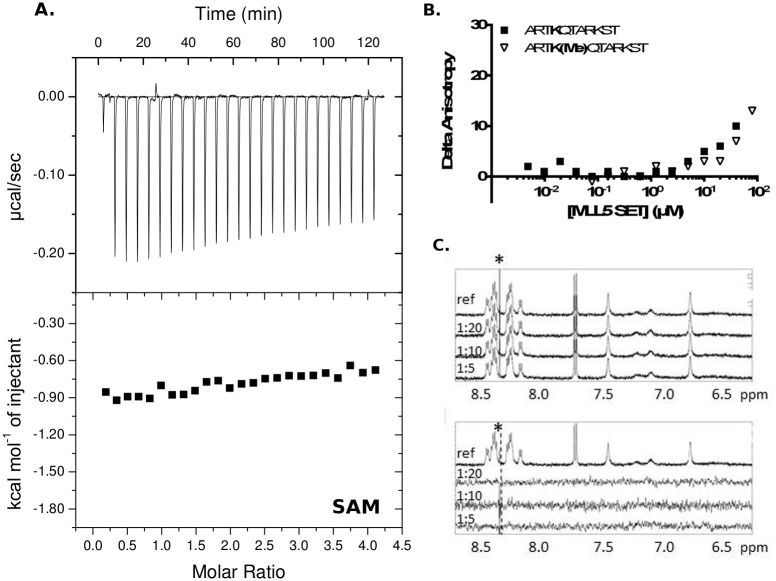
Absence of cofactor and substrate (H3K4 peptide) binding to the MLL5 SET domain. **(A)**. Isothermal titration calorimetry shows that the MLL5 SET domain in combination with the N-terminal flanking helix and the POSTSET does not bind the cofactor S-adenosyl methionine (SAM). Results were the same with the fragment SET-POSTSET. **(B)**. Fluorescence anisotropy shows that the MLL5 SET domain (SET-POSTSET construct) does not bind either the non-methylated or mono-methylated H3K4 peptide. **(C)**. ^1^H NMR spectra of SET-POSTSET construct and un-methylated H3K4 peptide. Upper panel: Expansion of ^1^H NMR spectra of un-methylated H3K4 peptide (top) at 400 μM, and in presence of different ratios protein/peptide (1:20, 1:10, 1:5). Lower panel: Expansion of ^1^H (top) and saturation transfer difference (STD) spectra of un-methylated H3K4 peptide at 400 μM in presence of different ratios of protein. In all spectra, the fine peak labeled with an asterisk (*), if present, corresponds to an impurity, whose resonance is imperfectly subtracted in STD on- and off-resonance experiments.

Although the MLL5 SET domain lacks some essential residues for methyltransferase activity, we investigated whether it was capable of interacting with the substrate or the cofactor SAM, two binding events necessary for catalysis. For these functional studies, we prepared a MLL5 fragment including the SET domain and the POSTSET region at the C-terminus (SET-POSTSET, residues 323–473) as well as a longer fragment containing an additional N-terminal helix (Nh-SET-POSTSET, residues 262–473), which was previously shown to be important for the structural integrity of the SET domain [13,16]. Isothermal titration calorimetry (ITC) measurements indicated that both fragments failed to bind the cofactor SAM (Fig 2A), and thermal denaturation shift assays revealed no stabilization of both SET fragments even in the presence of 20-fold molar excess of SAM (data not shown), thus confirming the ITC results.

Similarly, we ran fluorescence anisotropy experiments to measure the affinity of the presumed substrate H3K4 peptide (ARTKQTARKST), with un-methylated or mono-methylated lysine 4 [7], for the SET-POSTSET fragment. These measurements clearly showed that the interaction between the MLL5 SET domain and both histone H3 peptides is extremely weak (Fig 2B).

To ensure that the MLL5/H3K4 peptide interaction was not inhibited by the fluorescein moiety attached to the N-terminus of the peptides, we conducted NMR titration and saturation transfer difference (STD) experiments to monitor the interaction between the SET-POSTSET fragment and the unlabeled non-, mono- and di-methylated H3K4 peptides. Titration and STD analyses were performed by increasing the protein/peptide ratio from 1/20 to 1/5 in a stepwise manner. As shown in Fig 2C (upper panel), the line widths and the position of proton resonances of the un-methylated peptide remained unchanged, even at the highest protein concentration, although in the case of a low affinity complex, any interaction with the protein would cause a small line width increase and/or a shift of some proton resonances in the peptide spectrum [17]. STD experiments (Fig 2C lower panel), which are very sensitive to the presence of low affinity complexes (Kd in the 10^-4^–10^-7^ M range) did not reveal any resonances of atoms from the peptide in interaction with the protein. Identical results were obtained with all forms of the H3K4 peptide.

As we did not observe any interaction of the MLL5 SET domain with non-, mono- or di- methylated H3K4 peptides, we sought to identify other histone substrates for the MLL5 SET domain using GST-pull down and a mixture of histones from calf thymus. As shown in S3 Fig, no binding could be observed between the GST-fused SET-POSTSET fragment and any component of the histone mixtures.

Taken together, our data indicate that the isolated MLL5 SET domain in combination with the POSTSET region fails to bind either the cofactor SAM or any histone substrate. In addition, the presence of the N-terminal flanking region is not helpful for the SAM binding.

### The MLL5 SET domain does not exhibit any methyltranferase activity

**Fig 3:**
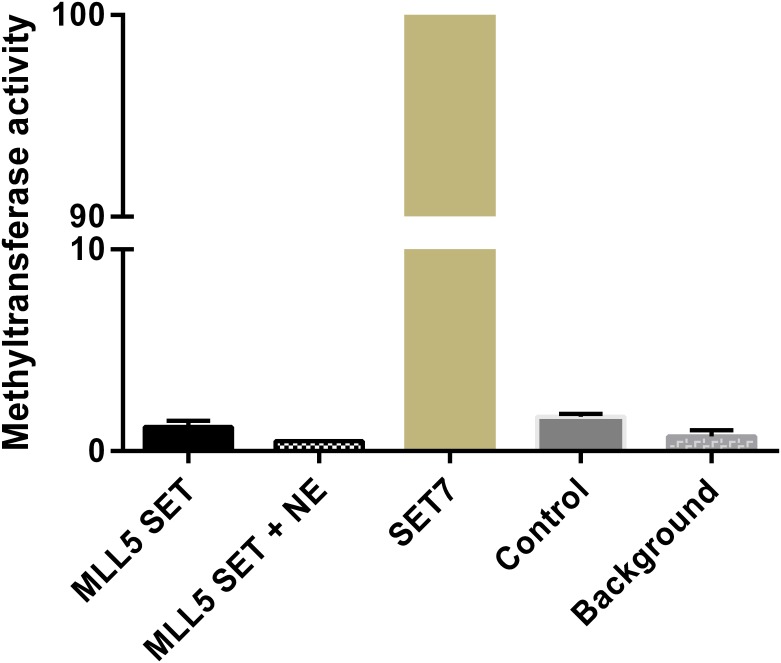
The MLL5 SET domain does not possess histone methyltransferase (HMT) activity. Radioactive HMT assays were performed with the bacterially purified Nh-SET-POSTSET fragment and histones from calf thymus as substrate. Recombinant proteins were incubated with HeLa nuclear extracts (+ NE) or not. SET7 was used as positive control (set as 100% activity) and the same condition in absence of any enzyme was used as negative control (Control).

Next, we performed an *in vitro* histone methyltransferase assay (HMT) to address whether the MLL5 SET domain is enzymatically functional. We followed the same protocol as the one used in the study of UpSET, the Drosophila ortholog of MLL5, showing that this SET domain does not exhibit histone methyltransferase activity [9]. We found that the bacterially expressed and purified Nh-SET-POSTSET fragment does not have HMT activity (Fig 3), either alone or in presence of nuclear extracts to allow the presence of additional proteins that could be necessary for a potential methyltransferase activity of MLL5. In fact, whereas the SET7 protein shows a strong methyltransferase activity as expected [18], the MLL5 fragment, alone or after incubation with mammalian nuclear extracts, exhibits a level of activity comparable to the negative control.

### The overall MLL5 SET domain structure

In order to better understand the molecular basis for the absence of HMT activity in the MLL5 SET domain, we solved its crystal structure. Attempts to crystallize the Nh-SET-POSTSET and SET-POSTSET fragments were unsuccessful. NMR studies (S4 and S5 Figs, S1 File) showed that both the N-terminal helix flanking region and the POSTSET region display substantial dynamics, but did not induce major changes in the structure of the SET domain. Such a flexibility, already observed in other SET-POSTSET proteins (like EZH2, [19]), would explain the difficulty to crystallize the longer fragments of the MLL5 SET-POSTSET domain.

**Fig 4:**
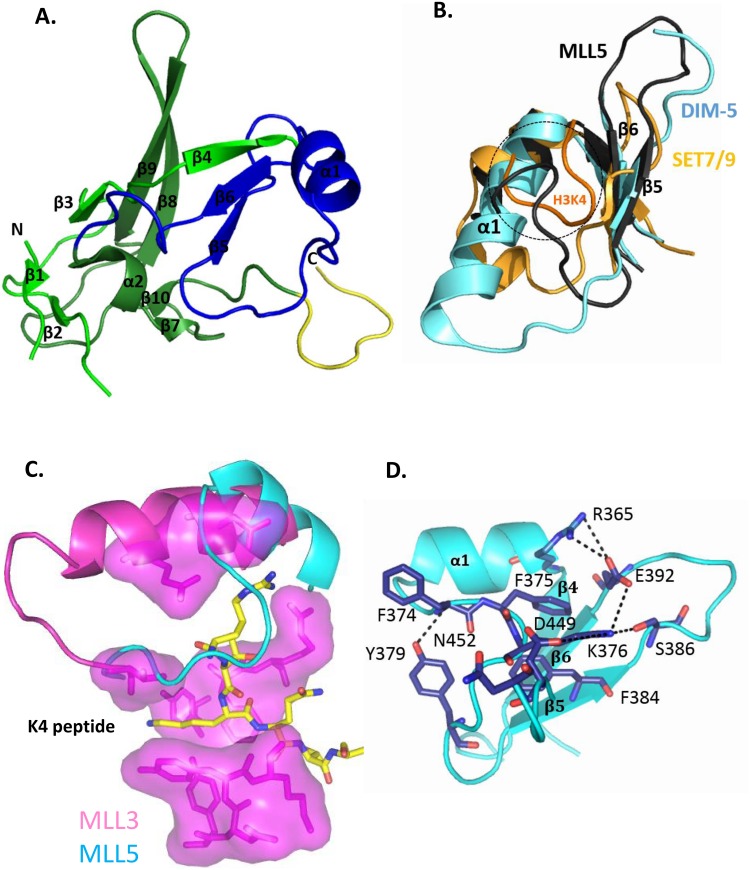
Crystal structure of the MLL5 SET domain highlights unexpected feature. **(A)**. The structure is represented as a cartoon model and secondary structure elements are labeled. The subdomains are: SET-N in pale green, SET-I in blue, SET-C in bright green and the beginning of the POSTSET domain in yellow. **(B)**. Part of the MLL5-SET (dark grey), DIM-5-SET (cyan, PDB entry 1PEG) and SET7/9-SET (light orange, PDB entry 1O9S) domains are superimposed and represented by cartoons. The histone peptide in complex with the SET7/9-SET-domain is colored in orange. The loop of the MLL5-SET domain occupies the region of the substrate binding groove (highlighted in a circle). **(C)**. Groove of K4 peptide binding of MLL3 (pink surface, PDB entry 5F6K) superimposed with the MLL5 structure (cyan) to highlight the occupation of the substrate groove by the MLL5 loop between α1 and β5. **(D)**. Zoom-out view of the interface (salt-bridge and hydrogen bonds as well as hydrophobic stacking between aromatic residues shown as dashed lines) that maintains the loop between α1 and β5 in the peptide groove.

The SET domain alone (residues 323 to 458) crystallized in the space group P3_1_21, and diffracted up to 2.1 Å resolution (Table 1, Fig 4A). To allow crystallization, we mutated an exposed and non-conserved cysteine (Cys 453) into alanine since this residue caused heterogeneity of the protein due to dimerization by formation of an intermolecular disulfide bridge. The crystal structure was determined by molecular replacement with many difficulties. First, most of the datasets collected could be integrated in the P3_1_21 space group with two different unit cell parameters (a = b = 66.2 Å, c = 56.7 Å, or a' = b' = 66.1 Å, c' = 113.3Å), due to the presence of a pseudo-translation along the c axis. A pool of 16 homologous SET models with less than 30% sequence identity was selected for initial molecular replacement trials. The best template models were then edited based on the local fit to the density maps to produce an ensemble of viable search models. Finally, by combining molecular replacement with this ensemble model in the large cell using Phaser-MR [20] and cyclic iterations of manual rebuilding with Coot [21] followed by auto-rebuilding with Buccaneer [22] and Parrot [23], we managed to generate an hybrid model resulting in a good molecular replacement solution (S6 Fig). The initial R / Rfree factors calculated after rigid body and restrained refinement steps with Refmac [24] were equal to 27% / 34%. The final crystal structure of the SET domain of MLL5 in its free form was refined at 2.1 Å resolution with an R-factor equal to 20.9% and R-free equal to 24.2% with good stereochemistry (Table 1).

**Table 1:** Data collection and structure refinement statistics.

	PDB entry 5HT6
**Data collection**	
Space Group	P3_1_ 2 1
Cell dimensions	
a, b, c (Å)	65.90, 65.90, 112.90
α, β, γ (°)	90.00, 90.00, 120.00
Resolution (Å)	50.93–2.09 (2.21–2.09)*
Rsym	0.06 (0.39)*
I/σI	5.87 (2.0)*
Completeness (%)	99.5 (99.7)*
**Refinement**	
Resolution (Å)	50.93–2.10
No. reflections	17250
Rfree test set	2432 (7.55%)*
Rwork/Rfree	0.209/0.242
No. atoms	
Protein	1931
Water	80
B-factors Å^2^	
Protein chains A/B	50.45/69.43
Water	46.79
R.m.s deviations	
Bond lengths (Å)	0.004
Bond angles (°)	0.841

* Values in parentheses are for highest-resolution shell.

The asymmetric unit contained two unliganded MLL5 molecules which superposed with a root mean square deviation (RMSD) of 0.64 Å (110 Cα atoms). Molecule A, which includes most of the SET polypeptide chain (residues 327–331, 340–457) was used for the structural analysis. Molecule B had well defined electron density for SET core, however residues 327 to 339 at the N-terminus and 449 to the end at the C-terminus were disordered and could not be modeled from X-ray data. However, the MLL5 POSTSET zinc binding site could be easily modeled using the MODELLER v9.2 program [25], and the homologous domain of the SETD2 methyltransferase (pdb entry 4H12) as a template [26] (S4 Fig, S1 File). This model suggests that, together with Cys 411 from the SET domain, the three conserved cysteine residues of the POSTSET region (Cys 459, 461 and 468) form a canonical zinc-binding site.

The overall tertiary structure of the MLL5 SET domain is similar to that of previously determined SET domain structures (Fig 4A). The RMSD calculated between backbone atoms of the core residues included in the SET-N (start to Leu 364), SET-I (Arg 365 to Gly 401) and SET-C (Asn 402 to Iso 433) sub-domains of the MLL5 and MLL1 SET domains is 1.3 Å (on 112 Cα atoms). The core of the SET domain is formed by a three-stranded (β3, β8, β9) anti-parallel β-sheet pressed diagonally across a second smaller one (β4, β5, β6). The opposite face of this smaller β-sheet is decorated by an α-helix (α1). A single-turn 3_10_ helix (α2) is packed against the core on the opposite side by one two-strand anti-parallel β-sheet (β1, β2) and one parallel β-sheet (β7, β10). Finally, a typical pseudo-knot is formed by the C-terminal segment of the SET domain passing through a loop formed by a preceding sequence stretch.

### The isolated MLL5 SET domain adopts a conformation that precludes SAM and substrate binding

A careful analysis of the crystal structure of the SET domain reveals several features that most likely account for the lack of catalytic activity of MLL5. A unique structural feature of the SET domain of MLL5 is a very short helix α1 (two turns, from Arg 365 to Asn 371) where other SET domains usually display a four-turn α-helix (e.g. DIM-5 and MLL3) (Fig 4B and 4C). None of the 3D prediction programs have anticipated this short α-helix, even if the presence of a helix-destabilizing residue (Gly 372) could explain the premature discontinuation of the helix.

**Fig 5:**
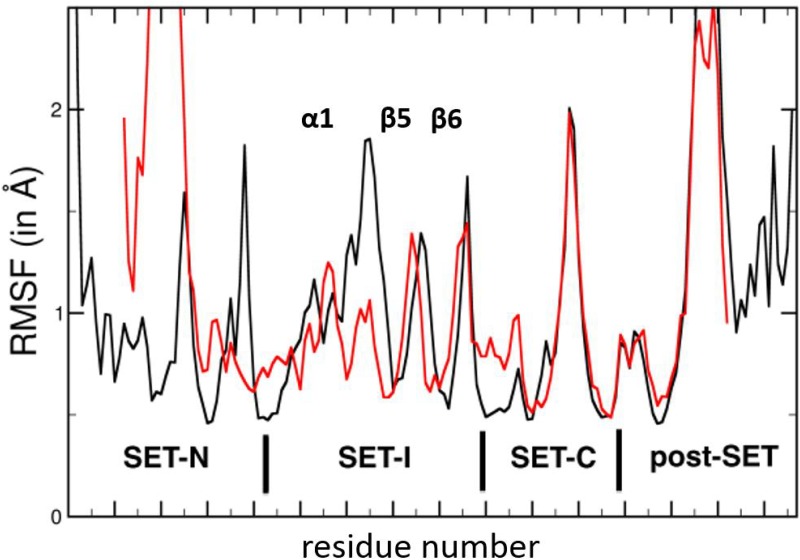
Dynamic properties of the unliganded MLL5 SET domain. The root mean square fluctuations (RMSF) of the SET domain residues during 100 ns of MD simulation are shown in red. The equivalent plot for the MLL3 SET domain isolated, taken from [16] and whose data where kindly sent by Y. Zhang, is shown in black.

Accordingly, the shortening of this helix results in the formation of a long loop (from Gly 372 to Phe 381) that is very uncommon in SET domains. This large loop between α1 and β5 occupies a groove which, in other SET domains, serves as a substrate docking site (Fig 4B and 4C). This groove normally mediates sequence-specific recognition and positions the substrate lysine side chain into a conserved channel that penetrates deep into the core of the SET domain (Fig 4C) and meets the cofactor at the active site (S1 Fig). In these SET domains, the long α1 helix forces the corresponding loop to pass outward of the SET domain and to delineate the substrate channel (Fig 4B and 4C). To our knowledge, only two other SET domains, from the methyltransferases SET7/9 and GLP, display such a short helix. However, contrary to MLL5, the elongated loops of these methyltransferases sit outside of the domain (Fig 4B). It is noteworthy that this MLL5-specific loop conformation is observed in both molecules of the asymmetric unit and does not rely on crystallographic constraints. In addition, residues from helix α1 and from the following loop create salt-bridge and hydrogen bonds with residues from the substrate channel (Fig 4D), maintaining it in this position. A measure of stability is the persistence of these hydrogen bonds and salt bridges throughout a molecular dynamics (MD) simulation. A 96.5% persistence over all frames of the trajectories is observed for the salt-bridge between Arg 365 and Glu 392 (S7 Fig), suggesting a limited flexibility of this region. In addition, 67% occupancy is detected for the salt bridge between Arg 376 and Asp 449, and an occupancy of 51.6% for the hydrogen bond between Lys 376 with Ser 386 and Asn 452. Our MD simulations also support the existence of a stable hydrogen bond between the phenolic hydroxyl group of Tyr 379 and the main chain nitrogen of residue 374, present for 83% of the simulation time and providing additional stability to the conformation of this loop segment. The calculation of root mean square fluctuations (RMSF) shows that the α1 helix and the following α1-β5 loop are much more constrained in MLL5 (Fig 5) than in apo-MLL1 and apo-MLL3 [16]. This suggests that the loop may not open up to accommodate the histone peptide. Moreover, normal mode analysis of apo-MLL5 did not reveal large low-frequency collective motion of the SET-I subdomain (data not shown), thus excluding local structural rearrangements of this loop. In conclusion, the elongation of the α1-β5 loop in MLL5 and its positioning in the peptide groove is very likely to preclude the binding of any substrate.

**Fig 6:**
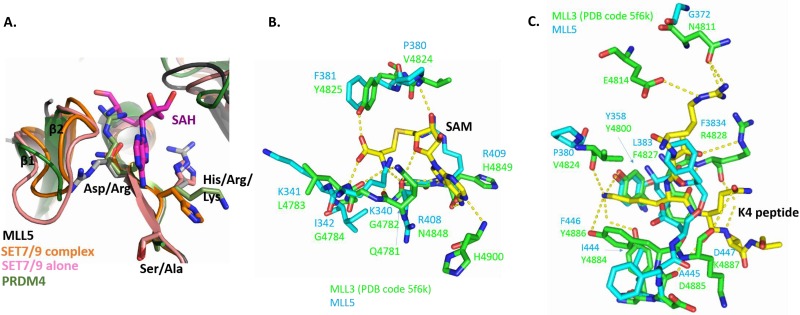
The S-adenosylmethionine (SAM) binding motif is not present and the active site is incomplete in the MLL5 SET domain. **(A)**. Superposition of the motif RRS of MLL5 SET domain (black and gray sticks), the motif NHS of SET7/9 SET domain in its apo (pink, PDB entry 1H3I) form and in ternary complex (orange, PDB entry 1O9S) and of the motif RKA of PRDM4 SET domain (green, PDB entry 3DB5). **(B)**. Residues of the MLL3 SET domain (PDB entry 5F6K, green sticks) forming hydrogen bonds with the cofactor SAM (yellow sticks). Corresponding residues of the MLL5 SET domain are superimposed (cyan sticks). **(C)**. Superimposition of MLL5 residues (cyan sticks) and the MLL3 active site (PDB entry 5F6K, green sticks). Hydrogen bonds stabilizing the histone peptide (yellow sticks) are shown as dashed lines.

Of note, the NH motif that is usually highly conserved in SET domains and necessary for the binding of the SAM cofactor is replaced by a RR (Arg 408-Arg 409) motif in MLL5 (Figs 1 and 6). The second arginine (Arg 409) is probably able to re-orient upon cofactor binding as illustrated by the reorientation of the corresponding residue (His 297) in SET7/9 (PDB entry 1H3I) upon substrate binding and in the presence of the cofactor SAH (PDB entry 1O9S) (Fig 6A). In the ternary complex, the histidine points towards the outside of the SET domain, a position that is also permitted for MLL5 Arg 409. On the other hand, the first residue of the motif (Asn 4848) of the MLL3 SET domain makes hydrogen bonds with the hydroxyl group of its side chain with three atoms of the cofactor SAM (Fig 6B). The replacement of this residue by an arginine (Arg 408) in the MLL5 SET domain precludes the formation of these hydrogen bonds (Fig 6B). In addition, this first arginine of the motif (Arg 408) in the MLL5 SET domain appears to prevent the proper positioning of the loop between the two first strands of the domain (β1 and β2). Indeed, the β1-β2 loop is disordered in the crystal structure, suggesting a high flexibility of this region. This loop which is well defined in both structures of SET7/9 in its free form and in the ternary complex [12] is necessary for the formation of a functional cofactor binding site. In PRDM4, for which no methyltransferase activity has been reported, the corresponding arginine residue (Arg 491) can also block the SAM binding site (PDB entry 3DB5) (Fig 6A). These results strongly suggest that the substitution, in the NH motif, of an asparagine by an arginine led to the loss of SAM binding by MLL5 proteins.

Regarding the catalytic center itself, the conserved Tyr 358 which favors the methyl transfer from the cofactor SAM to the histone peptide in other HMTs such as the MLL1-4 proteins [13,16] appears to be favorably positioned in MLL5 (Fig 6C). In contrast, all others residues important for peptide binding through hydrogen bonds in MLL3 and other MLL proteins are not conserved in MLL5 (Fig 6C). In particular, two tyrosine residues (Tyr 4884 and Tyr 4886 in MLL3), whose hydroxyl group form hydrogen bonds with the side chain of the lysine 4 of the histone peptide in the MLL3 structure complex, are replaced, respectively, by Ile 444 and Phe 446 in MLL5, both of which do not allow the formation of such hydrogen bonds (Fig 6C). In conclusion, the MLL5 SET domain lacks essential residues for the correct binding and positioning of both cofactor and substrate.

## Discussion

### Human MLL5 has no intrinsic HMT catalytic activity

The data presented here indicate that the isolated and bacterially expressed SET domain of MLL5, either alone or in combination with a complete POSTSET sub-domain, is not capable of binding either the cofactor SAM or the histone substrate and thus completely lacks histone methyltransferase activity. Even if we could not exclude a contribution of post-translational modifications in eukaryotic organisms, this absence of catalytic activity can be explained by several factors. First, the MLL5 protein lacks essential residues for cofactor and substrate binding. Interestingly, comparison of the human MLL5 SET sequence with those of orthologs from various mammalian species indicates that the complete MLL5 SET domain is strictly conserved (not shown). Therefore, the selective pressure on residues within the putative SAM and histone binding sites is comparable to that on structural residues, which suggests that the results obtained here on the human MLL5 protein can likely be applied to all mammalian MLL5 sequences. Second, structural analysis and molecular dynamics simulations of the MLL5 SET domain reveal the presence of a large loop between α1 and β5 that stably folds back to the protein core and prevents histone peptide binding. Previous studies described the reduction or lack of catalytic activity for isolated SET domains due to the incorrect positioning of structural elements around the substrate binding groove [13, 26, 27]. For example, in the isolated MLL1 SET domain, the peptide binding cleft is too wide, resulting in the suboptimal positioning of the substrate and a subsequent loss of catalytic activity [13]. In contrast, the EZH2 SET domain structure reveals a closed conformation that prevents peptide binding [26,27]. The recent crystal structure of the human PRC2 complex [28] allow to show that a re-orientation and stronger stabilization of the EZH2 SET-I in the complex permit the methyltransferase activity of EZH2. To our knowledge, MLL5 represents the first SET domain structure with such an occupation of the peptide binding cleft by a part of the protein. In conclusion, the MLL5 SET domain, in addition to lacking catalytic activity, is further characterized by an absence of H3K4 docking capability. A recent study suggests that, in human MLL5, this latter function is achieved by the PHD domain [29].

### Human MLL5 is not a member of the MLL family

Phylogenetic analyses of human, *Drosophila*, yeast and amphioxus sequences demonstrated that MLL5 and SETD5 belong to the same family, and that this family is clearly distinct from the MLL1-4 and SETD1A, B group. Contrary to MLL5, the SET domains of the MLL1-4 proteins have all the required catalytic residues and the methyltransferase activity of these proteins is well described [4,13,16]. In the case of MLL1, the relative positioning of the SET sub-domains is not optimal and its association of with the WDR5 (WD-repeat protein-5) complex is required for full activation. Likewise, the association of MLL3 and MLL4 with the RBBP5 (Retinoblastoma-binding protein-5)—ASH2L (Absent Small Homeotic disks-2-like) heterodimer reduces the inherent flexibility of their SET domains, favoring the formation of a catalytically competent conformation [16]. Interaction of these MLL proteins with a tri-subunit complex composed of WDR5-RBBP5-ASH2L involves a WIN (WDR5 interacting) motif in close proximity of the SET domain [30]. While conserved in MLL1-4 as well as in SETD1A, B, MLL5 lacks a WIN interaction motif [31]. In addition, Zhou and colleagues have recently shown that the WDR5, RBBP5 and ASH2L proteins are absent from the MLL5 complex [32]. Comparison of our MLL5 SET structure with that of the MLL3-RBBP5-ASH2L complex confirms that MLL5 does not have the residues required for interacting with this complex [16]. Instead of Val 4809 and Thr 4803 found in MLL3, MLL5 has bulky residues (Gln 367 and Lys 361, respectively) that are unable to accommodate the shallow pocket formed by RBBP5 and the SET domain. Based on our phylogenetic, structural, and functional analyses, it is thus very clear that MLL5 is not a member of the MLL family.

### What is the biological role for the human MLL5 SET region?

Although MLL5 lacks HMT activity, the presence of members of the MLL5/SETD5 family in various species (including, humans, cephalochordates, *Drosophila* and yeast) nonetheless suggests that they have important functions in the organism that are independent of the catalytic site. In addition, given the high level of sequence identity between SETD5 and MLL5, it is conceivable that they are functionally redundant, which might explain the relatively mild phenotypes observed in MLL5 knockout mice [5].

Instead of catalyzing histone methylation, recent studies point to a role of MLL5 in the regulation of the expression of other histones-modifying enzymes, like LSD1 or SET7/9. For example, Zhou and colleagues recently showed that MLL5 knockdown cells are characterized by a reduced H3K4 trimethylation rate at E2F1 target promoters, suggesting that MLL5 associates with other proteins (like HCF-1) to indirectly stimulate methylation [32]. Furthermore, human MLL5 as well as its *Drosophila* and yeast orthologs, UpSET and SET3, contain a PHD domain that strongly and specifically binds to histone H3K4me3 through a non-canonical mechanism [7,29,33]. Like human MLL5, *Drosophila* UpSET and yeast SET3 also have no HMT activity, but are found in multi-protein complexes exhibiting deacetylase activity [8,9]. Interestingly, the yeast SET3 complex (termed Set3C) shows similarities to the mammalian HDAC3 complex, involving the corepressors SMRT and N-CoR [8].

In conclusion, we hypothesize that MLL5 functions as a mediator of gene regulation in humans, not through a HMT activity, but as a structural component ensuring multi-protein complex integrity. Thus, MLL5 could have been retained in the genomes of a wide variety of organisms because of its capacity to engage in protein-protein interactions.

## Materials and Methods

### Phylogenetic analyses

Amino acid sequences included in the analysis are listed in S1 Table. Amino acid alignments were based on conserved protein domains identified using a NCBI CD-Search [34]. All alignments were performed using MUSCLE as implemented in MEGA7 [35], followed by manual refinement. All positions containing gaps and missing data were hence eliminated. In total, two separate phylogenetic analyses were performed, one using only human HMT proteins and one including HMT sequences from humans (*Homo sapiens*), the cephalochordate amphioxus (*Branchiostoma floridae*), the fruit fly (*Drosophila melanogaster*) as well as the yeast (*Saccharomyces cerevisiae*). The first tree was calculated with 24 sequences and a total of 105 amino acid positions, while the second one was obtained based on 22 sequences and a total of 206 amino acid positions. The phylogenetic relationships were assessed using both the Maximum Likelihood (ML) and Bayesian inference (BI) methods. For the ML analyses, two different phylogenetic models were used: the human HMT tree was calculated with the Whelan And Goldman + Freq. model [36] and a discrete Gamma distribution to model evolutionary rate differences among sites (5 categories) and the tree containing several species was computed with the Le and Gascuel model [37] and a discrete Gamma distribution to model evolutionary rate differences among sites (5 categories) with the rate variation model allowing for some sites to be evolutionarily invariable (+I). The robustness of each node of the resulting trees was assessed by Bootstrap analyses (with 1000 pseudoreplicates) [38]. All ML analyses were conducted using MEGA7 [35]. To validate the obtained ML results, trees were also computed using the Bayesian Inference (BI) method, as implemented in MrBayes version 3.1.2 [39]. BI phylogenies were obtained using the WAG model with a discrete Gamma distribution for amino acid substitutions [36] in two runs of 1,000,000 generations with sampling of trees every 100 generations and a burn-in period of 25%. All trees are drawn to scale, with branch lengths indicating the number of substitutions per site.

### Cloning, expression and purification

The human MLL5 cDNA was a gift from Shigeaki Kato (University of Tokyo, Japan). A fragment from this cDNA encoding the SET domain (residues 323–458) was sub-cloned into the pOPINJ vector from Ray Owens's lab [40], allowing the expression as a His_6_-GST fusion protein (named SET). In order to limit dimerization of the protein, a mutation of cysteine 453 into an alanine was introduced using the QuikChange site-directed mutagenesis lightning kit (Stratagene) (named SETC453A). This mutant was used only for crystallization purpose. Two longer constructs including the POSTSET domain were prepared by sub-cloning a fragment encoding residues 323–473 (named SET-POSTSET) and residues 262–473 (named Nh-SET-POSTSET). These two latest constructs do not contain the C453A mutation. All the proteins were expressed in Rosetta (DE3) *E*. *coli* cells. Cultures were grown overnight at 20°C after induction with 0.5mM IPTG. The cell lysate was first applied to a nickel affinity column (HiTrap HP, GE Healthcare) washed in 50mM Tris, HCl pH 7.5, 150mM NaCl and 1mM DTT. After cleavage of the GST-tag with PreScission protease on the column, a final purification step was added consisting in a gel filtration on a Superdex 75 column (GE Healthcare) equilibrated in the same buffer. 20μM of ZnSO_4_ was added in all purification buffers for the constructs that include the POSTSET domain. Fractions containing the protein of interest were pooled and concentrated to 8 mg/ml. The intact mass of the purified protein was confirmed by ESI mass spectrometry.

### Crystallization, data collection and structure determination

The protein SETC453A was crystallized by sitting drop vapor diffusion using a 1:1 drop ratio against a mother liquor containing 0.1 M MES pH 6.5 and 25% (w/v) PEG 3000. Crystals around 100 μm in size, were cryo-cooled by immersion in liquid nitrogen using 25% glycerol as cryoprotectant. Datasets were collected at the ID23-1 beam-line at the European Synchrotron Radiation Facility (Grenoble, France) and processed and scaled with XDS and XSCALE [41]. For molecular replacement, model rebuilding and refinement, a number of programs in CCP4 [24] and PHENIX [42] were used in combination (Phaser [20], Buccaner [22], Parrot [23], COOT [21], Refmac [43] and phenix.refine [44]).

### Nuclear Magnetic Resonance (NMR) experiments

The following protein buffer was used for NMR experiments: 50 mM Tris pH 7.5, 150 mM NaCl, 1 mM DTT (addition of 20 μM ZnSO4 for the SET-POSTSET protein). All spectra were recorded on a 500 MHz Bruker Advance spectrometer equipped with a cryogenic H/C/D/N probe with a Z-axis gradient at 300 K. For 1D titration experiments, series of 1D Saturation Transfer Difference (STD) NMR spectra [45] were recorded on different mixtures of isolated MLL5 SET-POSTSET protein and several forms of H3K4 peptide (un-, mono- or di-methylated). The peptide concentration was typically 400 μM and the peptide:protein ratio was systematically varied from 20:1 to 5:1. STD spectra were acquired with 2048 scans and selective saturation of MLL5 SET-POSTSET resonances at -0.5 ppm (-30 ppm for reference spectra). Saturation transfer of 2 s was achieved with equally spaced 50-ms Gaussian shaped pulses (separated by a 1-ms delay). The most shielded resonance of peptide is at 1.11 ppm, but an irradiation test was also performed on a free peptide sample to verify that only MLL5 SET-POSTSET resonances were irradiated. Subtraction of free induction decay values with on- and off-resonance protein saturation was achieved by phase cycling. A relaxation delay of 2.5 s (Aq + *D*_1_) and 128 dummy scans were employed to reduce subtraction artifacts. All NMR Spectra were processed and analyzed using Gifa [46] and Topspin 2.6 (Bruker Biopsin, Germany).

### Isothermal Titration Calorimetry (ITC)

The ITC experiment was carried out at 20°C on a MicroCal VP microcalorimeter. The Nh-SET-POSTSET protein sample was buffer exchanged by overnight dialysis into the ITC buffer (50 mM Na phosphate pH 7.5, 150 mM NaCl). Titration of MLL5 Nh-SET-POSTSET domain (30 μM) in the cell (2 mL) was performed by sequential addition of SAM (600μM, 25 injections of 10 μL). Integrated raw ITC data were fitted to a one binding site model using nonlinear least squares regression analysis with the MicroCal Origin plugin after the control experiments (titration of the SAM from the syringe into the buffer) were subtracted.

### Fluorescence-based thermal shift assays (Thermofluor^®^)

Solutions of 15 μL containing 40 μM of MLL5 SET-POSTSET protein, 2X Sypro^®^ Orange (Sigma) and different concentrations (ranging from 2 to 20 molar excess of protein) of SAM, S-adenosyl homocysteine (SAH) or histone peptide in 50 mM Tris-HCl pH 7.5, 150 mM NaCl, 1 mM DTT were added to the wells of a 96-well PCR plate. The plates were sealed with an optical sealing tape (Bio-Rad) and heated in an Mx3005P Q-PCR system (Stratagene) from 25°C to 95°C at 1°C intervals. Fluorescence changes in the wells were monitored with a photomultiplier tube. The wavelengths for excitation and emission were 545 nm and 568 nm, respectively.

### Fluorescence anisotropy measurements

The binding affinities of fluorescein-labeled H3 peptides (ARTKQTARKST with K4 un- or mono-methylated, purchased at EZBiolab) for MLL5 SET-POSTSET protein in the presence of SAM (at a concentration of 10 molar excess of the higher protein concentration) were measured using a Safire microplate reader (TECAN) with the excitation wavelength set at 470 nm and emission measured at 530 nm at room temperature. The following buffer solution was used for this experiment: 50 mM Tris HCl pH 7.5, 150 mM NaCl, 20 μM ZnSO_4_, 2 mM β-mercaptoethanol, 10 mM MgCl_2_, 10% glycerol.

### Histone methyltransferase assay

GST-MLL5 Nh-SET-POSTSET (1 μg) protein was incubated with or without HeLa nuclear extracts (200 μg at 5.0X10^9^, Cilbiotech) at 4°C in buffer (50 mM Tris pH 7.5, 150 mM NaCl, 0.5% NP-40, 12.5 mM MgCl_2_, 1 mM β-Mercaptoethanol, 10 μM ZnSO_4_). 24 hours later, 5 μg of bulk histones from calf thymus (Roche) and 1 μl ^3^H-SAM (15 Ci/mM, Perkin Elmer) were added for 90 min at 30°C. Methylation assay with SET7 was used as a positive control by mixing 5 μg of histones with purified Set 7 (1 μg) in 20 mM Tris-HCl pH 8.0, 4 mM EDTA, 1 mM PMSF, 0.5 mM DTT, and 1 μl ^3^H-SAM (15 Ci/mM, Perkin Elmer) for 90 min at 30°C. Methylation was measured by liquid scintillation assay as described [47]. After the incubation period, 10μl of the reaction mixture were spread onto Glass Microfibre filters GF/A (Whatman, GE Healthcare, Life Sciences). Filters were washed with 50 mM NaHCO_3_ (pH 9.0) and air dried for 2 hours. The amounts of methylated products were quantified by liquid scintillation on a Liquid Scintillation Analyzer Tri-Carb 2900 TR (Beckman Coulter).

### Molecular Dynamics (MD) simulations

The initial model used for all-atom MD simulations corresponds to the MLL5 molecule A in the asymmetric unit. The disordered missing loop connecting residues 331 and 341 in the N-terminal segment was modeled using MODELLER [48]. The protein was solvated in TIP3P water molecules [49] and neutralized in 150 mM NaCl salt. The MD simulations of the solvated proteins were performed using NAMD2 [50] with the CHARMM27 force field [51]. The protein was initially minimized and then heated to 310 K with harmonic restraints added during the first 250-ps equilibration steps. A 2-ns unrestrained simulation was sufficient to achieve equilibration. All simulations were performed with periodic boundary conditions using the NPT micro-canonical ensemble with pressure set to 1 atmosphere and temperature set to 310 K. The pressure and temperature were maintained using the Nose-Hoover Langevin piston and the Langevin thermostat respectively. The van der Waals interactions were calculated using a switching distance of 10 Å and a cutoff of 12 Å. The particle mesh Ewald (PME) method [52] was used to calculate long range electrostatic interactions, with a grid spacing of 1 Å. The total number of atoms was approximately 23,300. All the production runs were performed with 2-fs time steps in conjunction with the SHAKE algorithm to constrain covalent bond lengths between heavy and hydrogen atoms [53]. Two independent 100-ns simulations were performed on an Intel Xeon cluster. Trajectory data from the simulations were recorded and analyzed in VMD [54]. Normal modes were calculated with the WEBnm@ program [55] using the default parameters and Cα atoms of the MLL5 protein. WEBnm@ uses the elastic network model and calculates the six lowest-frequency vibrational modes (modes 7–12).

### Accession code

The atomic coordinates and structure factors have been deposited in the Protein Data Bank under the accession code 5HT6.

## Supporting Information

S1 Fig Comparison of the active center of MLL1 (PDB code 2W5Z) and MLL3 (PDB code 5F6K) complex structures. MLL3 residues (PDB entry 5F6K, green sticks) that form hydrogen bonds with the cofactor SAM (yellow sticks) and the K4 histone peptide (yellow sticks). Corresponding residues of the MLL1 SET domain are superimposed (PDB entry 2W5Z, pink sticks).
(TIF)

S2 Fig Phylogenetic analysis of human histone methyltransferase (HMT) proteins. **(A)**. Maximum Likelihood (ML) tree of human HMT proteins, with Bootstrap percentages (ranging from 0 to 100) as branch support indicated at each node. Branch lengths are representative of sequence substitution rates. **(B)**. Bayesian Inference (BI) tree of human HMT proteins, with posterior probabilities (ranging from 0 to 1) as branch support indicated at each node. Branch lengths are representative of sequence substitution rates.
(TIF)

S3 Fig No histone binding by the MLL5 SET domain. GST pull-down shows that MLL5 SET domain does not bind either histone H3 or a mixture of histones from calf thymus.
(TIF)

S4 Fig Influence of the POSTSET of MLL5 SET on its structure. **(A)**. Model of the MLL5 SET-POSTSET fragment. The MLL5 POSTSET zinc binding site was modeled using the MODELLER v9.14 program (32), and the homologous POSTSET domain of SETD2 methyl transferase (pdb entry 4H12) (50) as template. **(B)**. Superposition of the ^1^H-^15^N HSQC spectra of MLL5 SET domain (blue) and MLL5 SET-POSTSET fragment (green) recorded at 500 MHz. The 2D heteronuclear experiments were recorded at 300K, with the two proteins at a concentration of 400 and 100 μM concentration respectively, in 50 mM Tris pH7.5, 150 mM NaCl, 1mM DTT. Peaks labeled with a red asterisk are present only in the spectrum of the SET domain, whereas peaks framed in a box are present only in the spectrum of SET-POSTSET fragment.
(TIF)

S5 Fig Influence of the extending N-terminal flanking region of MLL5 SET on its structure. Superposition of the ^1^H-^15^N HSQC spectra of MLL5 SET-POSTSET domain (green) and Nh-SET-POSTSET (purple) recorded at 500 MHz. The 2D heteronuclear experiments were recorded at 300K, with the two proteins at a concentration of 100 mM concentration, in 50 mM Tris pH7.5, 150 mM NaCl, 1 mM DTT.
(PDF)

S6 Fig Models from the different steps of rebuilding. Superposition of the final MLL5 SET domain structure (same colors as in Fig 4A) with one of the best model used for molecular replacement (PDB entry 3BO5, colored in brown) and with the intermediate hybrid model (colored in red). The two regions whose modeling was crucial are circled.
(TIF)

S7 Fig Distance calculations suggest a persistent salt-bridge Arg 365-Glu 392 during MLL5 MD simulation. Plot of the distance between Arg 365 and Glu 392 (in red) and, as comparison, of the distance between Lys 376 and Asp 449. Asp 449 is localized in the POSTSET flexible segment. These values correspond to the distance between the center of mass of the oxygens in the acidic side chain and center of mass of the nitrogens in the basic side chain. The cutoff distance between the oxygen atoms of acidic residues and the nitrogen atoms of basic residues is 3.2A.
(TIF)

S1 File Comments on S4 and S5 Figs and supplementary methods. (PDF)

S1 Table Amino acid sequences included in the phylogenetic analysis. (PDF)
