# Supplementary figures and images for: The Human Mixed Lineage Leukemia 5 (MLL5), a Sequentially and Structurally Divergent SET Domain-Containing Protein with No Intrinsic Catalytic Activity
Source: PLoS One. 2016 Nov 3;11(11):e0165139. doi: 10.1371/journal.pone.0165139 (PMC5094779; doi:10.1371/journal.pone.0165139)

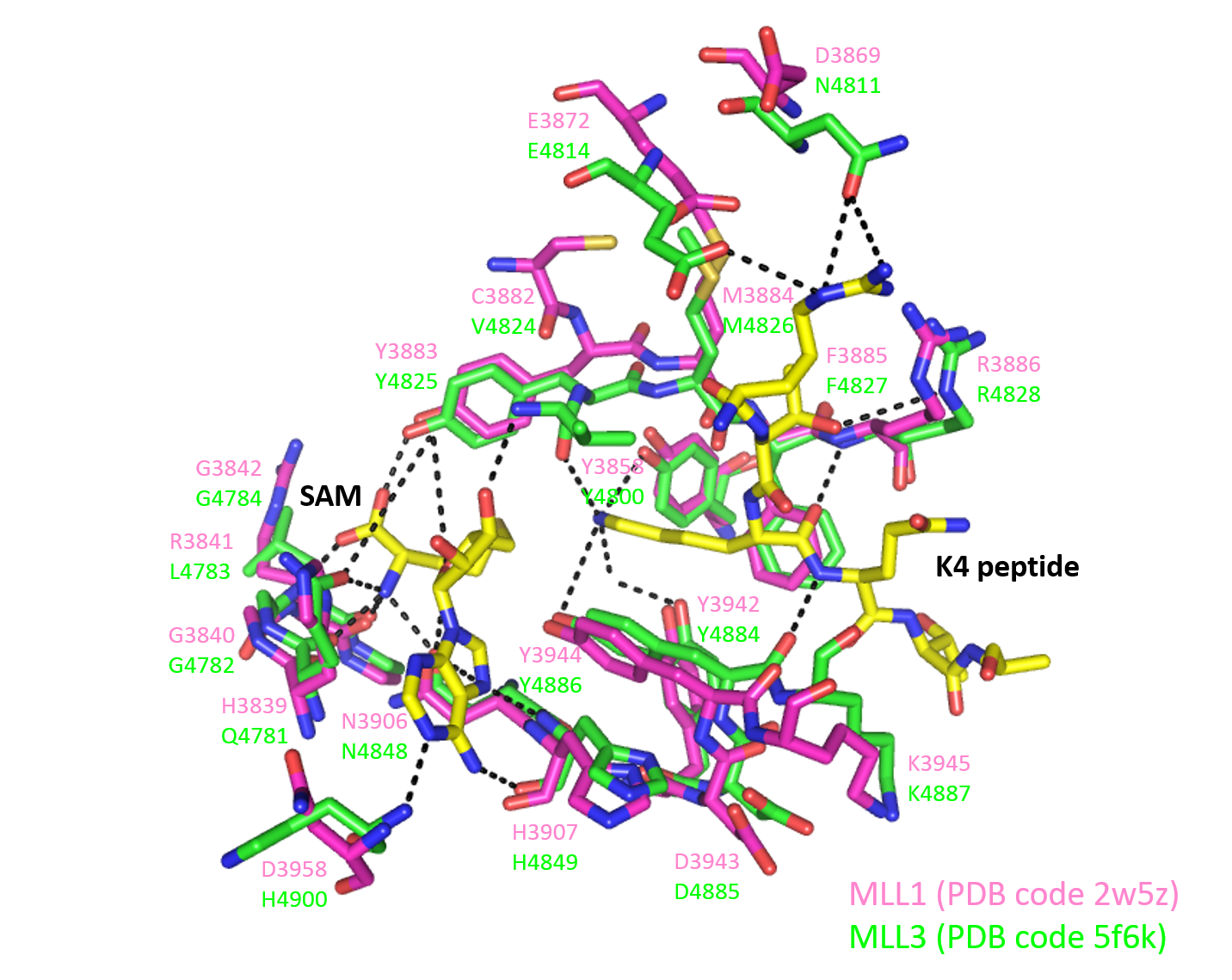

Supplement: S1 Fig — MLL3 residues (PDB entry 5F6K, green sticks) that form hydrogen bonds with the cofactor SAM (yellow sticks) and the K4 histone peptide (yellow sticks). Corresponding residues of the MLL1 SET domain are superimposed (PDB entry 2W5Z, pink sticks). (TIF) [file pone.0165139.s001.tif]

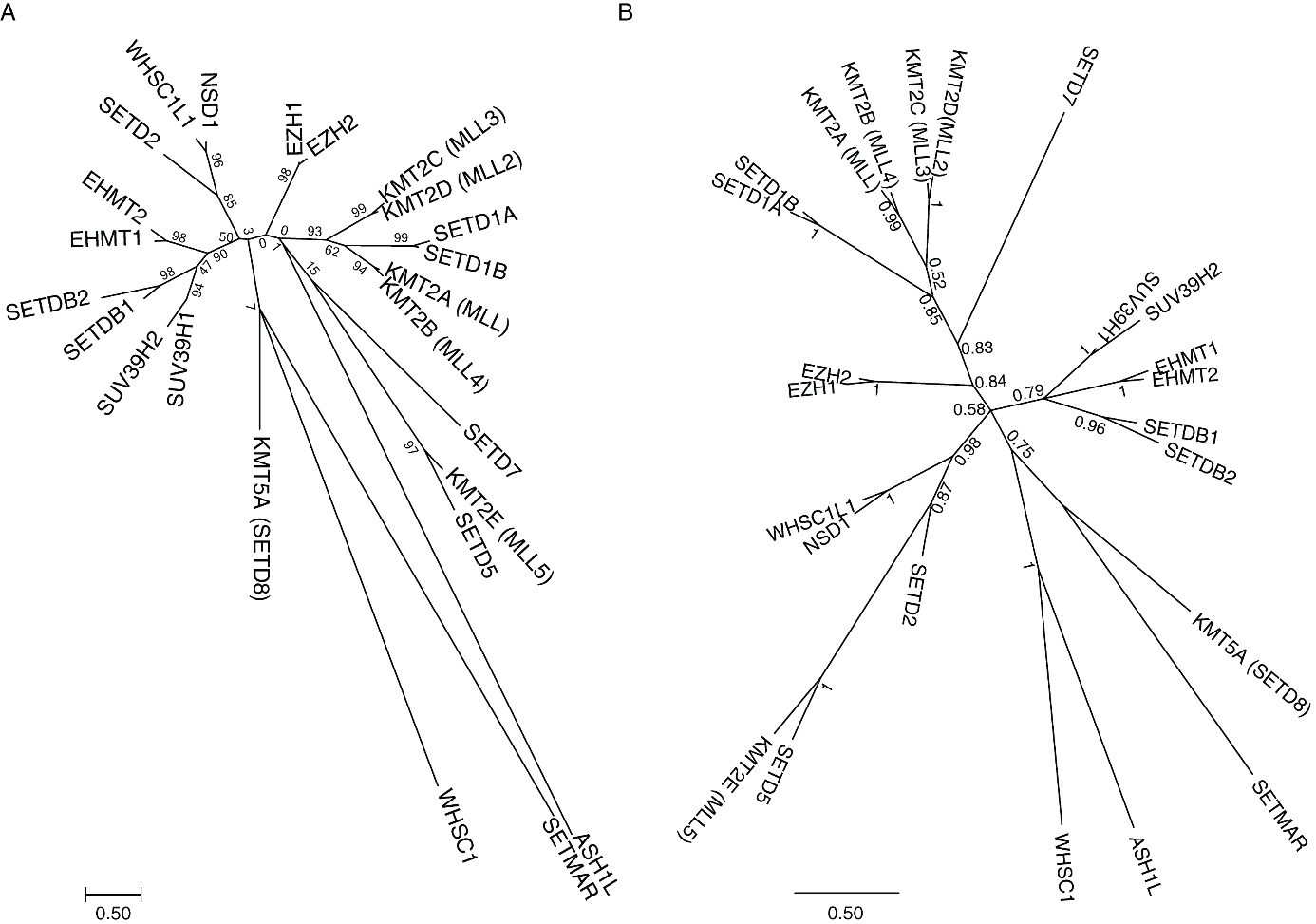

Supplement: S2 Fig — (A). Maximum Likelihood (ML) tree of human HMT proteins, with Bootstrap percentages (ranging from 0 to 100) as branch support indicated at each node. Branch lengths are representative of sequence substitution rates. (B). Bayesian Inference (BI) tree of human HMT proteins, with posterior probabilities (ranging from 0 to 1) as branch support indicated at each node. Branch lengths are representative of sequence substitution rates. (TIF) [file pone.0165139.s002.tif]

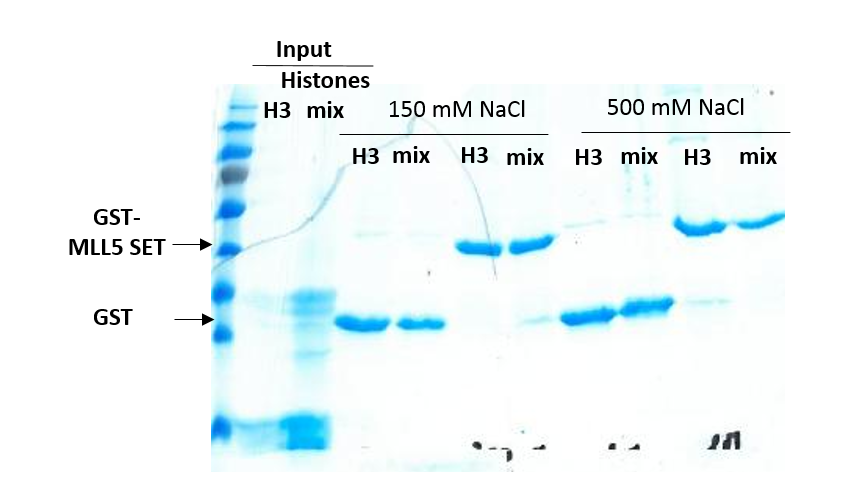

Supplement: S3 Fig — GST pull-down shows that MLL5 SET domain does not bind either histone H3 or a mixture of histones from calf thymus. (TIF) [file pone.0165139.s003.tif]

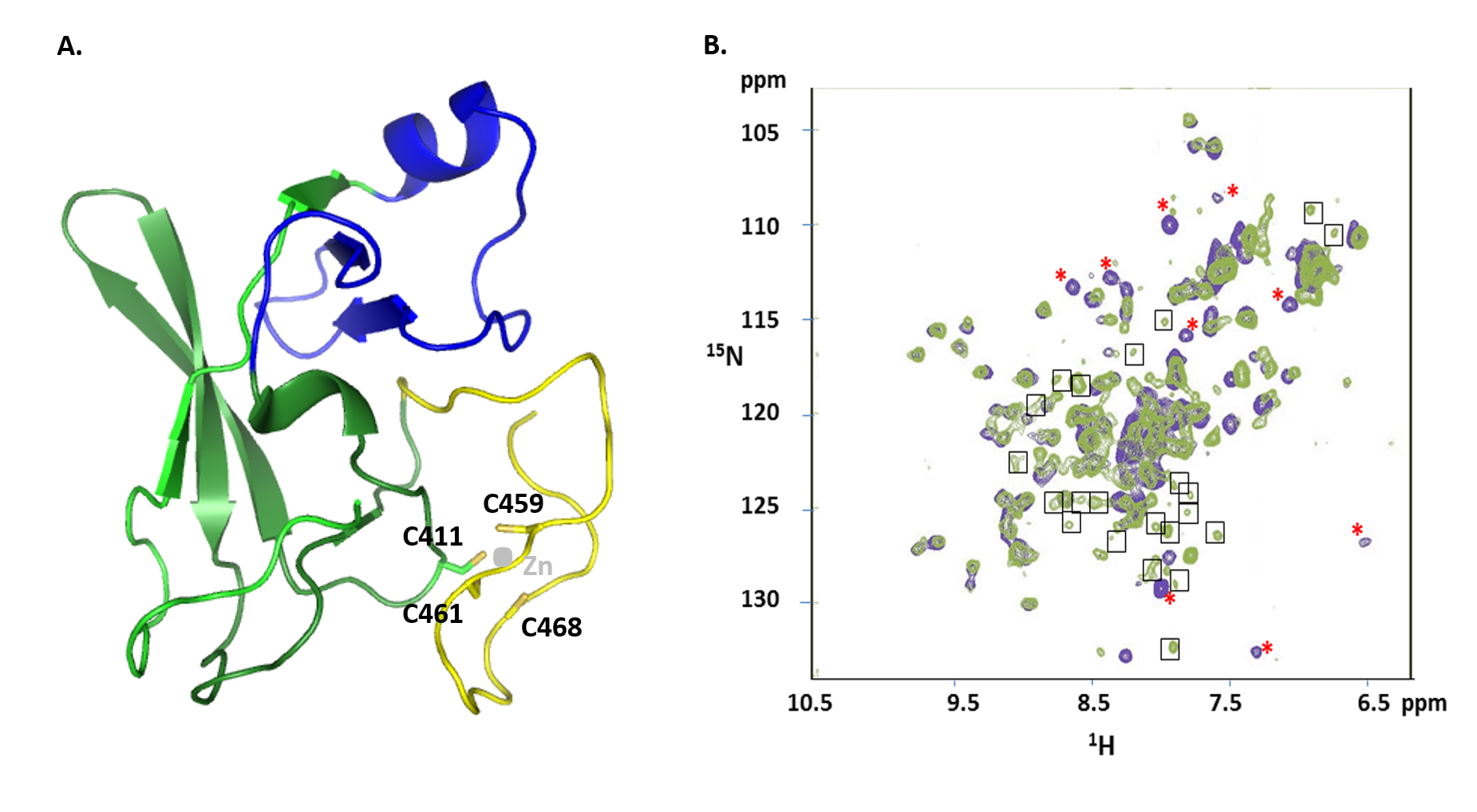

Supplement: S4 Fig — (A). Model of the MLL5 SET-POSTSET fragment. The MLL5 POSTSET zinc binding site was modeled using the MODELLER v9.14 program (32), and the homologous POSTSET domain of SETD2 methyl transferase (pdb entry 4H12) (50) as template. (B). Superposition of the 1H-15N HSQC spectra of MLL5 SET domain (blue) and MLL5 SET-POSTSET fragment (green) recorded at 500 MHz. The 2D heteronuclear experiments were recorded at 300K, with the two proteins at a concentration of 400 and 100 μM concentration respectively, in 50 mM Tris pH7.5, 150 mM NaCl, 1mM DTT. Peaks labeled with a red asterisk are present only in the spectrum of the SET domain, whereas peaks framed in a box are present only in the spectrum of SET-POSTSET fragment. (TIF) [file pone.0165139.s004.tif]

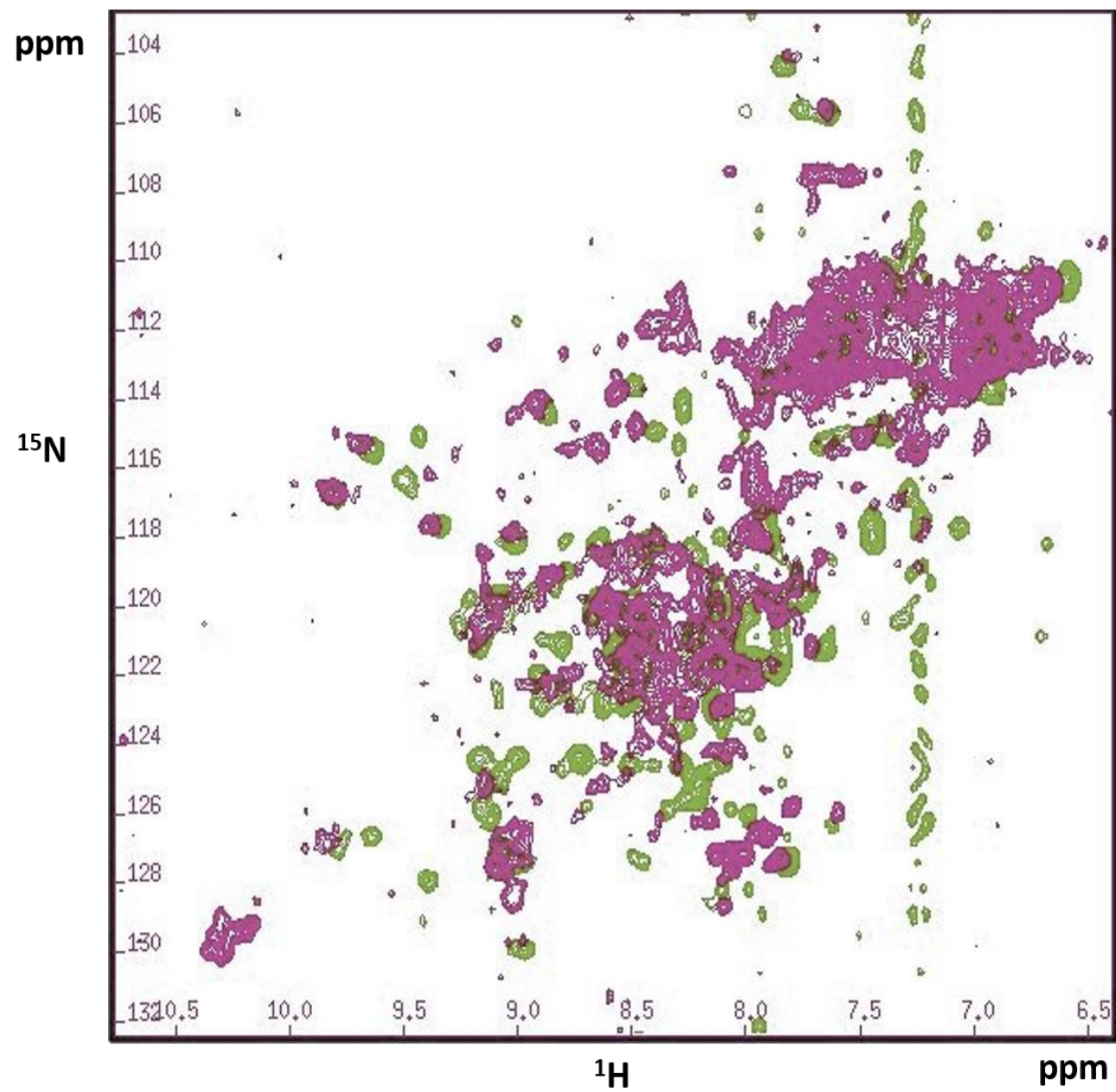

Supplement: S5 Fig — Superposition of the 1H-15N HSQC spectra of MLL5 SET-POSTSET domain (green) and Nh-SET-POSTSET (purple) recorded at 500 MHz. The 2D heteronuclear experiments were recorded at 300K, with the two proteins at a concentration of 100 mM concentration, in 50 mM Tris pH7.5, 150 mM NaCl, 1 mM DTT. (PDF) [file pone.0165139.s005.pdf]

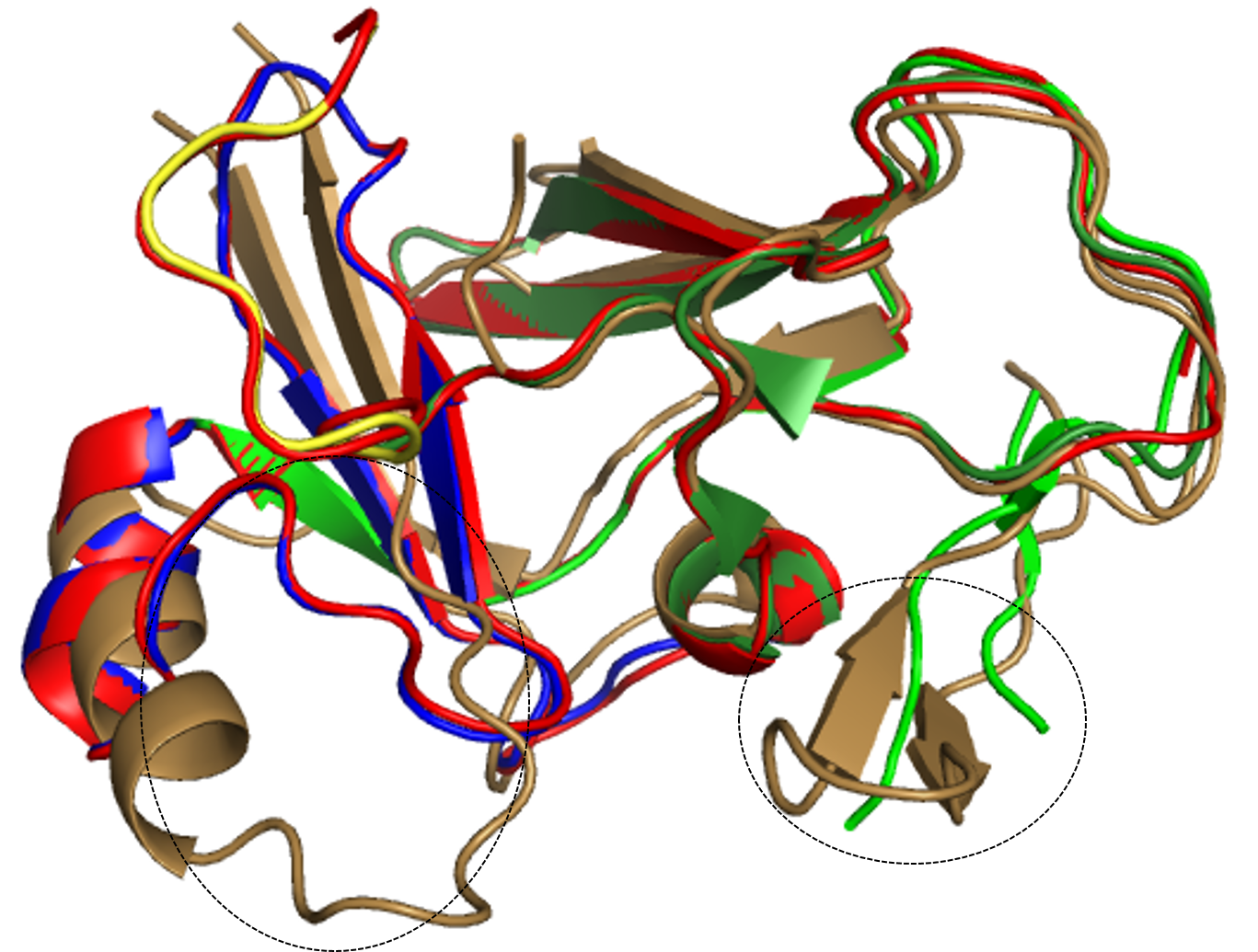

Supplement: S6 Fig — Superposition of the final MLL5 SET domain structure (same colors as in Fig 4A) with one of the best model used for molecular replacement (PDB entry 3BO5, colored in brown) and with the intermediate hybrid model (colored in red). The two regions whose modeling was crucial are circled. (TIF) [file pone.0165139.s006.tif]
