# Supplementary material for: The Human Mixed Lineage Leukemia 5 (MLL5), a Sequentially and Structurally Divergent SET Domain-Containing Protein with No Intrinsic Catalytic Activity
Source: PLoS One. 2016 Nov 3;11(11):e0165139. doi: 10.1371/journal.pone.0165139 (PMC5094779; doi:10.1371/journal.pone.0165139)
Supplement: S1 File — (PDF) [file pone.0165139.s008.pdf]

Comments of S4 Fig

As the two proteins differ only by 15 residues (including two prolines), similar spectral features were expected if their structures differ only marginally. Indeed, the two ^{1}H-^{15}N HSQC spectra superpose very well (Supplementary Figure 4B). However, the spectra show less peaks than expected. Around 100 out of 137 and 105 out of 150 were observed for the MLL5 SET domain alone and for the SET-POSTSET fragment, respectively (N-terminal hexahistidine tag and proline residues were excluded). Such a discrepancy between the number of expected and present cross-peaks indicates that both proteins exhibit substantial dynamics at the NMR timescale. The remaining peaks are well scattered, showing that they correspond to the folded parts of the proteins. Indeed, more than 30 of them have proton chemical shifts higher than 8.6 ppm, a low field range characteristic of amide protons implicated in β strands, in agreement with the predominance of this secondary structure element observed in the crystal structure. In order to retrieve more peaks, spectra were also recorded at pH 5.8 to lower the exchange rate of amide protons with water, but the resulting spectra were of poorer quality.

Interestingly, there are only a few peaks differing between the ^{1}H-^{15}N HSQC spectra of the SET domain and SET-POSTSET fragment of MLL5, suggesting that the adjunction of the POSTSET does not induce major changes in the structure of the SET domain. We found 20 peaks present only in the spectrum of the longer construct. Most of them are just above the noise level. Hence, if they belong to the POSTSET domain, it would indicate that this short segment exhibits also substantial dynamics at the NMR timescale. Such flexibility would explain the difficulty to crystallize the SET-POSTSET fragment. By contrast, nine peaks present in the spectrum of the isolated SET domain, most of them being rather intense, are not retrieved in the spectrum of the longer construct. Finally, a few peaks in the region between 9 and 9.5 ppm have broader line widths in the spectrum of the longer construct compared to the SET domain alone, suggesting that the POSTSET might somehow change the dynamics of the SET domain.

Comment of S5 Fig

There are only two tryptophan residues in the sequence, whereas four peaks are visible for these amino acids in the ^{1}H-^{15}N HSQC spectrum of Nh-SET-POSTSET. In addition, a number of peaks present in the spectrum of the SET-POSTSET protein disappeared or were highly shifted upon addition of the N-terminal helix region, while only a limited number of new

peaks appeared in the longer construct. Altogether, these NMR data suggest that the N-terminal region next to the SET domain is very flexible and significantly changes the dynamics of the SET domain probably by interacting with it.

Supplementary methods

GST pull-down experiment

Bacteria-expressed GST or GST-MLL5 SET-POSTSET proteins were immobilized on glutathione-sepharose 4B beads (GE Healthcare). Immobilized GST-tagged protein were incubated with a mix of histones from calf thymus (Roche), in presence of 20 molar excess of SAM in a binding buffer consisting in 50 mM Tris HCl pH 7.5, 150 mM or 500 mM NaCl, 10 mM DTT, 20 µM $ZnSO_4$ and 0.05% Tween20, for few hours at 4°C. The beads were then washed with the binding buffer and the bound proteins were separated by SDS-PAGE and stained with coomassie blue.

Nuclear Magnetic Resonance (NMR) experiments

^{15}N labeled proteins were purified from cells grown in minimal media M9 containing ^{15}N-NH_4Cl as the sole nitrogen source. The protein buffer was 50 mM Tris pH 7.5, 150 mM NaCl, 1 mM DTT (addition of 20 µM ZnSO4 for the SET-POSTSET protein to help the correct folding of the POSTSET). All spectra were recorded on a 500 MHz Bruker Advance spectrometer equipped with a cryogenic H/C/D/N probe with a Z-axis gradient at 300 K. ^{1}H-^{15}N HSQC correlation spectra were recorded using a water flip-back selective pulse and in States-TPPI mode. Spectral widths in ω1 and ω2 were 6493.5 Hz and 1520.5 Hz respectively, centered at 119.20 ppm in the ^{15}N dimension. ^{15}N decoupling was performed with a GARP4 sequence. Typically, between 128 and 140 complex points in the indirect dimension with 64-256 scans per FID were recorded. Total collection time varied between 2 hours and 12 hours, depending on the sample concentration. All NMR Spectra were processed and analyzed using Gifa (30) and Topspin 2.6 (Bruker Biopsin, Germany).
