# Supplementary material for: The Human Mixed Lineage Leukemia 5 (MLL5), a Sequentially and Structurally Divergent SET Domain-Containing Protein with No Intrinsic Catalytic Activity
Source: PLoS One. 2016 Nov 3;11(11):e0165139. doi: 10.1371/journal.pone.0165139 (PMC5094779; doi:10.1371/journal.pone.0165139)
Supplement: S1 Table — (PDF) [file pone.0165139.s009.pdf]

Table 1

Species	Sequence name	Accession number	Database
Homo sapiens	EHMT1	ENSP00000417980	Ensembl GRCh38.p5
	EHMT2	ENSP00000379078	
	EZH1	ENSP00000466924	
	EZH2	ENSP00000320147	
	KMT5A (SETD8)	ENSP00000384629	
	NSD1	ENSP00000395929	
	SETD2	ENSP00000386759	
	SETD7	ENSP00000274031	
	SETDB1	ENSP00000271640	
	SETDB2	ENSP00000346175	
	SETMAR	ENSP00000373354	
	SUV39H1	ENSP00000337976	
	SUV39H2	ENSP00000346997	
	WHSC1	ENSP00000329167	
	WHSC1L1	ENSP00000313983	
	ASH1L	ENSP00000357330	
	KMT2A (MLL)	ENSP00000436786	
	KMT2B (MLL4)	ENSP00000398837	
	KMT2C (MLL3)	ENSP00000262189	
	KMT2D (MLL2)	ENSP00000301067	
	KMT2E (ML5)	ENSP00000312379	
	SETD1A	ENSP00000262519	
	SETD1B	ENSP00000474253	
	SETD5	ENSP00000384114	
Branchiostoma floridae	ASH1L	124382	JGI v1.0 Brafl1
	KMT2A/B	278042	
	KMT2C/D	125138	
	KMT2E/SETD5	131096	
	SETD1A/B	125552	
Drosophila melanogaster	ASH1	FBpp0297151	FlyBase r6.10
	TRX	FBpp0082409	
	TRR	FBpp0070347	
	UPSET	FBpp0075510	
	SET1	FBpp0112592	
Saccharomyces cerevisiae	SET1	YHR119W	SGD S288C
	SET3	YKR029C	
	SET4	YJL105W	
